# Editorial: Women in brain-computer interfaces

**DOI:** 10.3389/fnhum.2023.1260479

**Published:** 2023-08-22

**Authors:** Zulay R. Lugo, Caterina Cinel, Camille Jeunet, Floriana Pichiorri, Angela Riccio, Selina C. Wriessnegger

**Affiliations:** ^1^Department of Neurology, University Hospital of Caracas, Caracas, Venezuela; ^2^Civil Association-Clinic Dispensary Padre Machado, Caracas, Venezuela; ^3^CSEE, University of Essex, Colchester, United Kingdom; ^4^UMR5287, Aquitaine Institute for Cognitive and Integrative Neuroscience (INCIA), Bordeaux, France; ^5^Santa Lucia Foundation (IRCCS), Rome, Italy; ^6^Institute of Neural Engineering, Graz University of Technology, Graz, Austria

**Keywords:** brain-computer interface, women, electroencephalography, disorders of consciousness, passive BCIs, neurofeedback

In this special Research Topic, *Women in brain-computer interfaces*, we intend to highlight recent research done or led by women in the field of brain-computer interfaces (BCI). As pointed out in the description of the topic, currently <30% of researchers in the world are women. Possibly some still persistent gender biases and stereotypes could be discouraging girls and women from pursuing a career in science, technology, engineering and mathematics (STEM) areas, all highly important fields in the development of BCI techniques.

In what we propose to be the first edition of an annual series dedicated to the topic of the role of women in BCI, several articles have been received fulfilling the fundamental requirement of the topic: a main authorship corresponding to women in order to highlight their involvement and interest in the field of BCI.

Forty years after the innovative idea proposed by Vidal of establishing a direct communication between the brain and computers in order to transmit and decode mental processes by means of electroencephalographic (EEG) signals allowing external actions (Vidal, 1973), technological development has allowed two parallel processes: on one hand, the development of electroencephalography (EEG) devices and computers with greater power, low cost and portability together with the appearance of new techniques such as functional magnetic resonance imaging (fMRI) and functional near-infrared spectroscopy (fNIRS) to be used in BCI research (Mridha et al., 2021).

On the other hand, we are observing the increasing survival rate of patients with severe brain injuries due to trauma or stroke, spinal cord lesions or progressive degenerative diseases, who require solutions to improve, restore or replace lost functions (McFarland and Wolpaw, 2011; Hochberg and Anderson, 2012; Rohm et al., 2013; Heilinger et al., 2018; Pichiorri and Mattia, 2020; Riccio et al., 2022). A more recent but already highly developed application of the BCI techniques is for the study of disorders of consciousness (DoC) and related states, to determine the presence of voluntary brain activity in apparently unconscious patients or to establish communication with conscious but severely brain-injured patients such as patients with locked-in syndrome (LIS) (Lesenfants et al., 2014; Gibson et al., 2016; Lugo et al., 2019). All these changes have widely expanded the BCI field of interest.

In the present Research Topic, Peters et al. highlight the wide use of BCI techniques in different types of lesions and diseases of the central and peripheral nervous system through a systematic review that describes the characteristics of the studies, systems and participants using augmentative and alternative communication BCI (AAC-BCI) systems.

Since EEG is the most widely used technique in BCI due to its mentioned low cost, easy handling and wide availability, there are many signals used and algorithms developed to improve the implementation of EEG-based BCI (Rashid et al., 2020). On this issue, Kostoglou and Müller-Putz describe in their article a new estimation method for cross frequency coupling (CFC), based on Linear Parameter Varying Autoregressive (LPV-AR) models, testing this method on both simulated and real data taken from patients with spinal cord injuries. This new approach might improve the decoding in motor related BCI applications for patients with this type of lesions.

Importantly, systems developed in the last 30 years of BCI research have become more and more interesting for a broader community of researchers. This can be due to improvements in the reliability and usability but also to the change of focus, namely from an application for disabled to healthy users (Blankertz et al., 2012). Especially the type of BCI, which are not consciously controlled by the user or reacting to external stimulation, called passive BCIs (pBCIs) are of main interest (Zander and Kothe, 2011). Having access to the user's ongoing brain activity enables applications spanning a variety of domains such as brain-activity based gaming (Bos et al., 2010; Holz et al., 2013), workload assessment (Wriessnegger et al., 2021); and neuromarketing (van Erp et al., 2012; Wriessnegger et al., 2017). In all these and other contexts and applications, feedback is a crucial aspect in the use of BCI techniques as it provides the subject with direct and continuous information on their own brain activity, thus allowing them to learn to modulate it in order to operate the BCI system, ultimately improving their performance (Sokunbi, 2017). Berger et al. report on explorations of a new type of visual feedback using 3D virtual reality in order to test if this type of paradigm may be more effective for the modulation of the sensory motor rhythm (SMR) than a 2D paradigm.

Finally, as already mentioned, a very important use of BCI techniques are its application in the study of DoC and related disorders as LIS, allowing to reconsider and reclassifying diagnoses. In this context, Schnakers et al. show how the detection of voluntary brain activity in non-responsive patients has led to the reconsideration of new nomenclatures for altered states of consciousness and Galiotta et al. performed a systematic review on the use of EEG-based BCI in patients with DoC. Both articles show the clear impact that t BCI techniques have had on the diagnosis, management and prognosis of these patients and also the challenges that these advances imply.

BCI is a vast field with multiple applications that pose both technical challenges and even ethical and legal considerations for its use (Chandler et al., 2022). We believe that woman can play a fundamental role in all aspects of BCI implementation and testing. As a highly multidisciplinary field, we hope that the BCI scientific community might lead the way toward gender equality in the nearby fields, e.g., STEM and neurorehabilitation among several to consider.

## Author contributions

ZL: Conceptualization, Writing—original draft, Writing—review and editing. CC: Supervision, Visualization, Writing—review and editing. CJ: Visualization, Writing—review and editing. FP: Conceptualization, Formal analysis, Supervision, Validation, Visualization, Writing—review and editing. AR: Conceptualization, Formal analysis, Supervision, Validation, Visualization, Writing—review and editing. SW: Validation, Visualization, Writing—review and editing, Conceptualization, Formal analysis, Investigation, Supervision.
